# A Web-Based Prostate Cancer–Specific Holistic Needs Assessment (CHAT-P): Multimethod Study From Concept to Clinical Practice

**DOI:** 10.2196/32153

**Published:** 2022-10-19

**Authors:** Veronica Nanton, Rebecca Appleton, Nisar Ahmed, Joelle Loew, Julia Roscoe, Radha Muthuswamy, Prashant Patel, Jeremy Dale, Sam H Ahmedzai

**Affiliations:** 1 Warwick Medical School University of Warwick Coventry United Kingdom; 2 Mental Health Policy Research Unit Division of Psychiatry University College London London United Kingdom; 3 Medical School University of Sheffield Sheffield United Kingdom; 4 Lucerne School of Business Lucerne Switzerland; 5 Prostate Cancer UK London United Kingdom; 6 Institute of Cancer and Genomic Sciences University of Birmingham Birmingham United Kingdom

**Keywords:** Holistic Needs Assessment, prostate cancer, survivorship, cancer follow-up, coproduction, web-based communication, care planning

## Abstract

**Background:**

Men with prostate cancer experience immediate and long-term consequences of the disease and its treatment. They require both long-term monitoring for recurrence or progression and follow-up to identify and help manage psychosocial and physical impacts. Holistic Needs Assessment aims to ensure patient-centered continuing cancer care. However, paper-based generic tools have had limited uptake within cancer services, and there is little evidence of their impact. With the expansion of remote methods of care delivery and to enhance the value of generic tools, we developed a web-based Composite Holistic Needs Assessment Adaptive Tool-Prostate (CHAT-P) specifically for prostate cancer.

**Objective:**

This paper described the context, conceptual underpinning, and approach to design that informed the development of CHAT-P, starting from the initial concept to readiness for deployment. Through this narrative, we sought to contribute to the expanding body of knowledge regarding the coproduction process of innovative digital systems with potential for enhanced cancer care delivery.

**Methods:**

The development of CHAT-P was guided by the principles of coproduction. Men with prostate cancer and health care professionals contributed to each stage of the process. Testing was conducted iteratively over a 5-year period. An initial rapid review of patient-reported outcome measures identified candidate items for inclusion. These items were categorized and allocated to overarching domains. After the first round of user testing, further items were added, improvements were made to the adaptive branching system, and response categories were refined. A functioning version of CHAT-P was tested with 16 patients recruited from 3 outpatient clinics, with interviewers adopting the think-aloud technique. Interview transcripts were analyzed using a framework approach. Interviews and informal discussions with health care professionals informed the development of a linked care plan and clinician-facing platform, which were incorporated into a separate feasibility study of digitally enhanced integrated cancer care.

**Results:**

The findings from the interview study demonstrated the usability, acceptability, and potential value of CHAT-P. Men recognized the benefits of a personalized approach and the importance of a holistic understanding of their needs. Preparation for the consultation by the completion of CHAT-P was also recognized as empowering. The possible limitations identified were related to the importance of care teams responding to the issues selected in the assessment. The subsequent feasibility study highlighted the need for attention to men’s psychological concerns and demonstrated the ability of CHAT-P to capture red flag symptoms requiring urgent investigation.

**Conclusions:**

CHAT-P offers an innovative means by which men can communicate their concerns to their health care teams before a physical or remote consultation. There is now a need for a full evaluation of the implementation process and outcomes where CHAT-P is introduced into the clinical pathway. There is also scope for adapting the CHAT-P model to other cancers.

## Introduction

### Background

The increased incidence of many common cancers, combined with rising survival rates, is challenging the ability of health services to meet the range of patient needs both during and after treatment. Various models of care delivery have been implemented to meet these escalating demands. Cancer services are currently focused on the potential of digital technology to facilitate innovations, particularly in terms of enabling remote patient follow-up and monitoring [1]. The COVID-19 pandemic has highlighted the value of such systems and provided a stimulus for their further rapid development and implementation.

Composite Holistic Needs Assessment Adaptive Tool-Prostate (CHAT-P) is a digital technology designed to contribute to innovation in care delivery. As a web-based, adaptive, cancer-specific needs assessment, it enables men with prostate cancer to easily identify and communicate their concerns to members of their health care teams. The output of CHAT-P can be shared across settings to facilitate care coordination. In addition, triggered by the concerns identified, it provides links to sources of advice and information. The structured output of CHAT-P allows patients’ priorities to drive the consultation, whether it is remote or face to face, and the tailored resources are designed to support self-management.

### Aims

This paper describes the context and rationale for the design of CHAT-P, its conceptual underpinning, and the process of development, from the initial concept to readiness for deployment, across 3 stages of development. Through this narrative, we sought to contribute to the expanding body of knowledge regarding the design process of innovative digital systems with potential for enhanced cancer care delivery.

### The Context: Prostate Cancer, Treatment Side Effects, and Follow-up

Prostate cancer is the most common cancer among men in the United Kingdom, affecting 1 in 8 over their lifetime (Prostate Cancer UK [PCUK]; [2]). With the 10-year survival rate at 78% [3], there are approximately 400,000 men in the United Kingdom living with or after the disease [4].

Treatments vary considerably for men within this patient population depending on factors such as disease stage, presence of comorbidities, and patient choice. Treatments may involve surgery, radiotherapy (using various delivery modes), androgen deprivation therapy (ADT), and chemotherapy for advanced disease [5]. Men identified to be at low risk with low-grade cancer may be offered active surveillance involving monitoring prostate-specific antigen (PSA) levels and regular contact with specialist clinicians. Men considered too frail for radical treatment and whose cancer does not present immediate problems may be assigned to *watchful waiting* before commencing ADT when symptoms occur.

Although survival rates are high, a diagnosis of prostate cancer has both immediate and long-term impacts on men’s lives. Each treatment type is associated with changes across various aspects of quality of life [6]. For example, in many cases, radical prostatectomy leads to urinary and sexual dysfunction that may persist long term [7], while men who have undergone radiotherapy are particularly at risk of developing bowel problems, which may also be long lasting [7]. The consequences of ADT include loss of libido, weight gain, gynecomastia, and cognitive effects such as memory impairment [8]. Finally, men on active surveillance and watchful waiting may experience increased levels of distress due to the uncertainty of an untreated cancer [9].

In addition to these direct effects, men experience indirect impacts of the illness and its treatment, such as effects on relationships [10], occupations, and finances [11,12]. Hence, the prevalence of depression and anxiety across treatment types and over time is relatively high in comparison with the general population, with peaks identified at certain critical points in the care pathway [13].

Men diagnosed with prostate cancer require lifetime monitoring. Men assessed as being at low risk following curative treatment will often also need ongoing care and support as well as monitoring to detect progression or recurrence. The use of digital systems for PSA tracking is increasing within UK cancer services, and the digital reporting of standardized patient-reported outcome measures for audit purposes is being trialed in prostate and other forms of cancer [14,15]*.* The recent National Prostate Cancer Audit (2021) recommendations R4 and R5 highlight the need to respond to men’s information needs regarding treatment and side effects and the importance of identifying and referring those who require specialist help for physical or psychological effects after treatment [16].

### Conceptual Underpinning: Holistic Needs Assessment

The concept of Holistic Needs Assessment (HNA) dates back several decades [17-21]. The concept of holism itself is underpinned by humanistic philosophy assuming a unity between the physical, psychological, social, and spiritual aspects [22]. In nursing, the recognition of the importance of a holistic approach to care has been traced back to its origins in the work of Florence Nightingale [23]. Person-centered care, now a central tenet of the National Health Service (NHS), builds upon the holistic understanding of the individual, acknowledging the primacy of the patient’s own values and the importance of their active participation in decision-making [24]. Although lacking an explicit theoretical underpinning, HNA is closely aligned with the patient-centered approach, recognizing the needs of the whole person.

In the United Kingdom, the assessment of holistic needs was first recognized in cancer care by the National Institute for Care and Excellence guideline of 2004 on supportive care for adults with cancer [25]. The value of HNA was again emphasized by the National Cancer Survivorship Initiative, as survival rates for several common cancers were improving and many patients were living for years with both the direct and indirect effects of the illness and its treatment. HNA typically involves a structured questionnaire that includes a range of domains, from physical symptoms and psychological issues to information needs and broader social, financial, and spiritual concerns. Its aim is to facilitate communication between the patient and the health care professional and to enable a course of action to be mutually determined and documented in a care plan [26]. One of the first HNA tools to be designed for use among cancer survivors and palliative care populations was the Sheffield Profile for Assessment and Referral for Care, which was developed by 2 of the coauthors of this study (NA and SHA) [27]. Originally paper based, a shift toward an electronic format for HNA has taken place in recent years.

The National Cancer Survivorship Initiative [28] recommended the use of HNA for patients with cancer to monitor unmet needs at key stages in the care pathway: following diagnosis, at the end of treatment, when positive or negative events occur, and at the transition to end-of-life care. However, the adoption of these principles has been slow and patchy throughout cancer services [29].

Resistance to HNA and its lack of impact may, in part, be explained by the generic format of the HNA instruments currently in use. This limits the extent to which HNA reflects concerns related to particular cancers. Moreover, the simple structure and limitations of using paper forms allow only the identification of high-level issues. A significant weakness of generic HNA in cancer is that it does not necessarily identify high-risk problems that may require urgent follow-up, many of which are specific to each cancer type. Thus, although generic HNA enables the selection of broad areas of concern, its value remains dependent on further identification of specific issues (which differ between cancer types) during the consultation before in-depth exploration.

### Approach to Design: Digital Technology in Prostate Cancer Care

The potential of digital technology to transform care and outcomes for men with prostate cancer was the focus of a global program of work initiated by the international nongovernmental organization the Movember Foundation in 2014 under the umbrella title of TrueNth [30]. In the United Kingdom, a range of projects were planned, coordinated, and overseen by the nongovernmental organization PCUK in a series of workshops involving men with prostate cancer, clinicians, and academics, which were delivered between 2014 and 2018. Discussions between patient representatives and researchers with experience in needs assessment and prostate cancer led to the concept of a prostate cancer–specific HNA that would be adaptive to the circumstances and needs of the individual patient. These discussions demonstrated that patients were frustrated by the limitations of follow-up consultations, which were perceived as being too narrowly focused and failing to address various needs such as concerns over the risk of their sons developing the cancer or the impact of their treatment on their relationships. This was attributed to time pressure and prioritization of monitoring for disease progression or recurrence.

Within the UK TrueNth program, the original purpose of the web-based prostate cancer–specific HNA was to provide men at low risk, whose care could be managed outside specialist services, with a PSA tracker system (estimated at up to 50% of those diagnosed) [31], which would identify their concerns to support workers. However, to make use of the full potential of adaptive digital technology to offer a broad as well as an in-depth assessment, the decision was made to create a system that would be suitable for all men with prostate cancer from diagnosis onward, regardless of the treatment pathway.

### Approach to Design: Coproduction

In health care, the concept of coproduction refers to collaborative approaches to intervention development where health care professionals and patients work together, drawing on both their expertise to create “new opportunities for innovation and involvement” [32]. Coproduction has been described as representing a shift in power toward the patient and a recognition of patients as experts in their own experiences [33].

Patient representatives and clinicians were involved in all stages of this project, from the initial identification of the issues the intervention sought to address and thereafter, playing an advisory role throughout development and testing. Several rounds of user testing took place during the 2-year development period before testing in a clinical setting. Each stage of development was further guided by a project steering group comprising patient representatives recruited through PCUK, urologists and an oncologist, researchers, and information technology specialists.

### Web-Based Platform: CHAT-P

**Figure 1:**
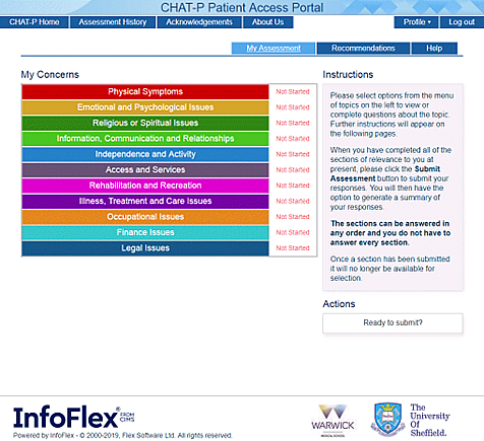
The 11 domains of Composite Holistic Needs Assessment Adaptive Tool-Prostate.

CHAT-P is a web-based adaptive system designed to be used by men remotely before (or potentially in some instances instead of) a face-to-face or remote consultation in secondary or primary care. It is divided into 11 different sections, each representing a particular domain (Figure 1), with men being able to choose sections of relevance to themselves and their preferred order of completion. The questions included in these sections are adaptive, meaning that the response to top-level screening questions could open further detailed questions if problems were identified or skipped if not. Once CHAT-P is complete and has been submitted, a summary of results is made available to a linked member of the patient’s health care team. This can be used as the basis for a care plan to address any identified issues or concerns. In addition, CHAT-P includes links to sources of reliable information and advice. A key feature of CHAT-P is that answers to specific questions identified by clinicians as needing urgent, rapid assessment (hereafter referred to as “red flags,” eg, blood in urine, unexplained lower back pain, and the feeling that life is not worth living) could trigger an immediate message to the nominated health care professional.

## Methods

### Overview

We used an iterative process involving scoping, development, and user testing, followed by refinement and testing with patients in a qualitative study. Further refinement took place following the qualitative study in conjunction with the completion of a clinician-facing site and care planning facility. CHAT-P development took place in 3 stages, which are described as follows: stage 1, the process whereby we conducted initial scoping and development; stage 2, the qualitative study used to test CHAT-P in a patient population; and stage 3, the development of the individualized care plan and clinician-facing site.

### Stage 1: Scoping of the Project, Generation of Item Bank and Domains, and User Testing (2013-2015)

The initial phase involved discussions between members of the project team, which included patient and public involvement representatives and members of the UK branch of the TrueNth Network, to clearly define the scope and scale of the project and identify its key requirements from the initial general concept.

The essential elements are summarized in Textbox 1.

**Key requirements of the web-based prostate cancer–specific Holistic Needs Assessment.**

**Requirements**

- Suitable for all men from diagnosis onward
- Menu driven with a branching system adaptive to individual concerns
- Links to sources of information and advice related to specific concerns
- Inclusion of “red flag” items

During the initial set-up and scoping phase, a survey on the use of HNA and associated problems and benefits in prostate cancer services in the United Kingdom and cancer information management systems was undertaken to inform the design and to preempt potential downstream barriers. In addition, a rapid review of patient-reported outcome measures and quality of life instruments relevant to prostate cancer, either specific or generic but used in the context of prostate cancer, was carried out to inform the design and content of the system (PCUK, unpublished reports, 2013, 2014).

A total of 61 assessment tools were identified during the review. The questions from these tools were inserted into a master item bank created in a Microsoft Excel spreadsheet with additional items identified by the expert group.

VN and NA reviewed all 2790 questions included in the item bank to derive high-level domains or categories for the structure of CHAT-P. An iterative process of developing domains and checking for the inclusivity of items led to the final classification of 11 broad domains. Each item was then assigned to a domain. Certain items were assigned to more than one category; for example, items concerning appetite were assigned to both physical and psychological concerns. The response categories from the 61 instruments were entered into a linked database. Question format and response categories were then developed for each item aimed at identifying the level of concern and were standardized into categorical verbal scores, for example, “none at all,” “a little bit,” “quite a bit,” and “very much,” reflecting the earlier Sheffield Profile for Assessment and Referral for Care tool [17]. In other domains, response categories from all the instruments were considered, and a choice was made or a new response category was assigned before acceptability testing with users. Certain domains, such as finance, legal, and information needs, did not require levels (how much and how often). The details of this process are provided in Multimedia Appendices 1 and 2.

To make full use of the potential of the digital system, the structure of CHAT-P was planned as both hierarchical and branched; the selection of top-level domains opened up menus leading to further questions depending on the items selected. This design was mapped out by NA and VN on paper and reviewed by the research team before the development of the prototype for user testing. Building of the web-based platform from the prototype to the final version of CHAT-P was undertaken by a commercial medical IT company, Infoflex-CIMS (now CIVICA, UK).

Next, 2 sessions of face-to-face testing of the CHAT-P prototype were held in different parts of the United Kingdom to capture the diversity of experience. In total, 10 men with prostate cancer, who were members of the PCUK, attended events at each location. Each participant was provided with a laptop and instructions on how to log in and navigate CHAT-P. The men were asked to complete a short questionnaire on their views on the value and purpose of HNA and specific questions regarding the domains incorporated, question and response format, font, and color scheme. This provided the starting point for a discussion in which men offered their views on the potential for care delivery and specific elements of the design that were priorities for the men in terms of usability and practicality of the system.

Following adjustments to wording based on the men’s suggestions and some improvements to the branching structure, a further round of user testing took place remotely with a total of 10 men from the original 2 workshops.

Finally, in this stage of development, links to sources of information and advice from quality-assured sources (eg, PCUK and Macmillan Cancer Support) were incorporated into CHAT-P to assist men in managing their concerns. These sources include web pages, video clips, or other documents. The appearance of the links is triggered by the selection of certain items that represent a current concern. This context-sensitive feature prevents men from being overloaded with irrelevant information.

### Stage 2: Testing With Patients (2016)

#### Overview

The first fully functioning version of CHAT-P was ready for testing with patients by early 2016. Here, we describe the conduct and findings of a nested qualitative study: “Holistic Assessment and Care Planning In Prostate Cancer.” This study explored the experience of using CHAT-P with men currently receiving treatment, monitoring, or follow-up for prostate cancer. The care planning facility was not a focus of this investigation, as its design was not completed at this time.

#### Aim and Objectives

The overarching aim of this stage was to understand patients’ perceptions of the potential of CHAT-P to enhance care.

We sought to explore (1) how men with a range of clinical characteristics and different levels of experience with IT responded to CHAT-P; (2) their views on its design, usability, and utility to themselves and others; (3) and their views on its limitations and barriers to its adoption.

#### Methodology

##### Design

The chosen design was a cross-sectional qualitative interview study.

##### Ethical Considerations and Governance

The interviews were designed with a focus on minimizing distress to participants, as we did not want to directly ask about sensitive topics. We followed the principles of co-design, where members of our steering group with lived experience were involved throughout. The study was approved by Yorkshire and the Humber Bradford Leeds Research Ethics Committee on February 2, 2015, and a substantial amendment was approved on June 6, 2016 (Research Ethics Committee reference: 15/YH0021).

The study was conducted in urology clinics in 2 NHS Foundation Trusts in central England. The sponsor of this study was the University of Warwick.

##### Eligibility

Men with a diagnosis of prostate cancer at any stage of disease or stage in the care pathway (other than those actively receiving specialist palliative care), the ability to give informed consent, and the ability to read English were eligible to participate.

##### Recruitment

Purposive sampling was used to recruit participants from different clinical settings. Eligible patients were selected from urology clinic lists at site 1 by clinical nurse specialists and a urology consultant surgeon. Study information packs containing letters of invitation and reply slips were sent to the patients. At site 2, a member of the research team (RA) visited a urology clinic and was invited by a clinic staff member to approach eligible patients to whom she explained the study and handed information packs. The study information packs were also sent by post to eligible patients on an active surveillance pathway. Interested participants returned reply slips to the research team, and interviews were scheduled.

Face-to-face interviews were conducted by trained researchers (RA and JL) either in the clinic or in the patients’ homes, audio recorded, and transcribed. The data were collected between October and December 2016.

##### Study Procedure

Written consent was obtained from the participants by the researcher before the interviews. Participants were introduced to CHAT-P, which was demonstrated to them, and the key features were explained. Men were then invited to select domains to try out for themselves.

Semistructured interviews took place while participants were trying CHAT-P using the “think-aloud” technique [34]. They sought to capture men’s views on the experience of using CHAT-P and their views on its potential for cancer care.

##### Analysis

The 2 data sets were combined for data analysis, which was performed using a framework approach [35]. This method produces structured data summaries, which facilitates coding and thematic development.

A total of 4 overarching categories were identified a priori to reflect the aims of this study, namely the user interface and design, suitability of content, personal value, and implementation and use in the clinical care pathway.

Following familiarization with the first 4 transcripts by 3 members of the project team, 1 team member assigned the data to the 4 categories and undertook initial coding. In addition, 2 other members of the research team scrutinized the coding and participated in an iterative process of developing further codes and subcategories until a complete analytical framework was derived. The remaining data were coded into the framework, which was then shared with the members of the wider project team. Themes, subthemes, and concepts were developed and refined through discussion and reflection to produce the final analysis.

## Results

### Overview

A total of 16 participants took part in the interviews, with 5 patients recruited from site 1 and 11 from the list of patients on active surveillance at site 2. The participant characteristics are presented in Table 1.

The findings relating to the 4 a priori categories (user interface and design, suitability of content, personal value, and implementation and use in the clinical care pathway) are described in subsequent sections, exploring men’s views on the use of CHAT-P in the prostate cancer care pathway and their feedback on the design and content. The findings within each theme are grouped into categories and illustrated using relevant participant quotations.

**Table 1:** Participant characteristics.

Study ID	Age band (years)	Treatment	Time since the start of treatment	Comorbidities	Self-reported computer literacy
D001	65-70	Not known	13 years	None mentioned	Yes—high
D002	65-70	Not known	—^a^	Hearing problems	Yes—medium
D003	65-70	Radiotherapy and hormone injections	13 years	None mentioned	Yes—high
D004	65-70	Not known	10 years	None mentioned	Yes—high
D005	80-85	Hormone treatment and radiotherapy	8 years	Chronic obstructive pulmonary disease, restricted mobility, and collapsed lung	Yes—high
B001	70-79	Active surveillance	—	None mentioned	Yes—medium
B002	70-79	Active surveillance	—	Arthritis	Yes—low
B003	50-59	Active surveillance	—	None mentioned	Yes—high
B004	50-59	Active surveillance	—	None mentioned	Yes—high
B005	70-79	Radical prostatectomy	2 years	Depression	Yes—high
B006	70-79	Active surveillance	—	Diabetes	Yes—high
B007	70-79	Radical prostatectomy	6 months	None mentioned	Yes—medium
B008	60-65	Active surveillance	—	Head and neck cancer (all clear)	Yes—low
B009	65-69	Radical prostatectomy	1 month	None mentioned	Yes—medium
B010	70-79	Active surveillance	—	None mentioned	Yes—medium
B011	70-79	Active surveillance	—	Hearing loss	No

^a^This information was not available for this participant.

### User Interface and Design

#### Ease of Use

Most participants found CHAT-P straightforward to use. The instructions were useful, comprehensive, and in the right font size:

I think it’s brilliant for those people that are reasonably computer literate, and even if they’re not [sic] I think that’s pretty easy to follow.

D001

However, 5 participants found that there was too much text on the introductory page with the instructions or that the font size was too small.

#### Graphics

The colors and images were received well by most participants, who commented that they liked the look of CHAT-P. One participant commented that the images should be enlarged. For example, the icons used to indicate a link to further information were overlooked by most participants before the researchers pointed them out.

### Suitability of Content

The participants were generally very satisfied with the content, with many commenting on the difficulty of creating a system responsive to the needs of patients with such diverse experiences.

#### Scope

The range of the questions also met with approval from the participants:

Well as I said it’s very comprehensive, it would be very difficult to try and think of anything that it hasn’t covered.

B005

There’s enough down there without being too much, but I think there’s enough down there that you’re covering everybody, or hopefully everybody.

B008

The participants also appreciated being able to select only the sections that were relevant to them once they understood the concept. All the participants saw some sections that were relevant to them or would have been at another stage of their care pathway:

I mean I know some of these things don’t probably apply...well they don’t apply to me because I mean I’m ten years down the line now, but generally most of these questions, at the time I would like to have the answer to anyway.

D004

I can see you’re trying to cover everybody.

B004

#### Improved Access to Web-Based Information

The participants valued the way in which the HNA can help make information that is readily available on the internet more accessible and make it easier to find what they needed:

But the fact that you’ve got them all together here, would make it a lot easier and a lot quicker to find the information you need. And all the information that I’ve got off the internet seems to be here.

B007

#### Questions on Comorbidities

Some participants questioned the relevance of the subsections and items about symptoms that are not directly related to prostate cancer, prompting the researcher to clarify the purpose of CHAT-P as an HNA designed to flag various concerns and issues, including comorbidities. Others deemed the inclusion of comorbidities very important:

I’ve got diabetes which affects some of my answers to questions. I’ve got other issues which affected my answers. So unless you’ve got a complete picture, or a reasonable picture, of the illness, the state of health of the people, then I think you’re probably missing out on a lot of answers.

B006

#### Sensitive Questions

The participants were asked whether some of the questions were too sensitive. All of them felt that the questions were appropriate, and a few mentioned that they had learned to talk about sensitive issues because of their condition and were comfortable in addressing such issues:

No, I’ve gone beyond being sensitive about personal things so it’s fine.

B009

### Personal Value

#### Identification and Articulation

The participants recognized the value of CHAT-P in helping them to think about concerns that they might not have considered otherwise:

As I see it at the moment it’s very comprehensive and it almost seems to be taking you down routes that perhaps you wouldn’t automatically have gone yourself, you know, things which may be due to other problems, nothing to do with the condition.

B005

...this is good in that it asks questions that I don’t even think about.

B006

In addition, the HNA was seen as a help to both articulate a wide range of needs and gain a more structured understanding of their needs; that is, being able to classify concerns:

I think to do it once is interesting because it enables myself, for instance, to think about things more fully than I would normally do. Or to think about things in little boxes, rather than as a global thing, which is what everybody does.

B006

#### Preparation for the Consultation

The participants also identified the potential of CHAT-P in preparing for a consultation by gathering information, thus enabling more informed communication with the clinician:

I would have liked to have used it myself at home. I think the more you know before you talk to your consultant or your doctor, the more you can understand about it. And that’s what I did. I mean, I went through YouTube and all sorts, to get as much information as I could so I knew what the consultant was talking about and what the options were. But as I say, if I’d have had this, it would have been a lot quicker and more clear.

B007

#### Preferences

For some, disclosure through a digital assessment was preferable to a face-to-face conversation. Others questioned whether they would want to disclose some mental health concerns to their clinician or whether they would feel embarrassed to admit the number of concerns they had.

### Implementation and Use in the Clinical Care Pathway

#### Use Over Time

The participants identified a variety of clinical settings in which CHAT-P could be used, including a hospital and general practitioner (GP) environment at times before, during, or after treatment. They also identified points in time that were particularly relevant, including after the diagnosis, when information needs are high in terms of treatment options, and immediately after treatment when care comes to an abrupt end:

I think if somebody has been diagnosed with prostate cancer from day one, I think once the, the news has sunk in, perhaps a couple of weeks later if they’re invited to go down to, I don’t know, the local GP or the nurse or something and say, well we know it’s early days but these are some of the issues that might arise, and do that exercise. And then perhaps repeat it, the same exercise sort of, I don’t know, six months later or a year later, that sort of thing, perhaps when they’ve had the treatment that they need.

D001

It’s the post op stage and knowing whether it’s been successful, what are the implications of the operation, so it’s round about that period where I think it’s most useful because you have all sorts of questions...

B005

#### Time Saving

Many participants mentioned that CHAT-P could save the clinician’s time by focusing a consultation on the patient’s key concerns and by providing information to the patient that they might otherwise have to gather elsewhere:

Yeah, I could see it will save them time, and also make it easier, because...when you come out of the consultation you think, oh I wish I’d said so and so.

B002

The biggest benefit I see of something like that is you save your clinicians time, because if it’s something that’s relatively straightforward that you find out your answer, you’re not going to be on the phone to them, seeing them unnecessarily. So it frees up more time, so it’s more treatment, and the whole thing becomes a lot more cost-effective.

B003

#### Acceptability to Service Users

The participants appreciated the “red flag” capability of CHAT-P to pick up critical issues and prompt patients to contact a health professional as well as the summary of patient concerns that is generated in the clinician-facing site. However, it was pointed out that to be of value, men needed to know that the concerns they identified through CHAT-P would be picked up and addressed by the relevant clinician:

So it’s having confidence I suppose, in the system, that if you’re basically putting stuff in from a question point of view...that one it’s being picked up, and secondly it’s being dealt with by the appropriate kind of person.

B003

Many participants expressed enthusiasm toward CHAT-P, anticipating its imminent availability. Some participants expressed an intention to use the system themselves, as illustrated by a patient on active surveillance:

I would be very keen on seeing this up and running. I think I’d find that a very, very useful tool that would save me hours and hours and hours of wandering about looking for various bits; it brings it all together. Yeah, I quite like that.

B005

### Summary of the Stage 2 Qualitative Study

The participants recognized the potential of CHAT-P to facilitate the communication they saw as central to their care. By enabling them to easily identify and articulate their concerns and access relevant information, the participants felt they would be better prepared and able to take an active role in the subsequent consultation. CHAT-P would enable clinicians to focus immediately on their specific concerns by providing a clear summary before or at the start of the consultation. However, the participants also reported that the value of CHAT-P would depend to a large degree on the clinician responding to the issues raised.

Feedback provided by the participants, which included the need for increased clarity in the user guidance, was collated and discussed with the study steering group. Further wording alterations and other small refinements, including changing the icons representing the internet links, were agreed upon and implemented.

### Stage 3: Development of Care Plan (2016)

Although further improvements to functionality continued, the focus of the final phase of development was the design, content, and format of the output of CHAT-P. This mainly comprised the care plan, which needed to be incorporated into a clinician-facing site. This stage was essential before testing within the clinical pathway. Interviews and discussions with clinicians, including urologists, oncologists, CNSs, and GPs, identified key features, which are outlined in Textbox 2. A total of 6 urologists provided comments collated through the TrueNth United Kingdom Supported Self-Management project colead. Other specialties were represented in the CHAT-P study steering group.

**Key features of the care plan.**

**Features**

- Concerns summarized on one page
- Red flag symptoms included
- Concerns presented in the order of importance
- Space for recording clinician and patient actions
- Downloadable

**Figure 2:**
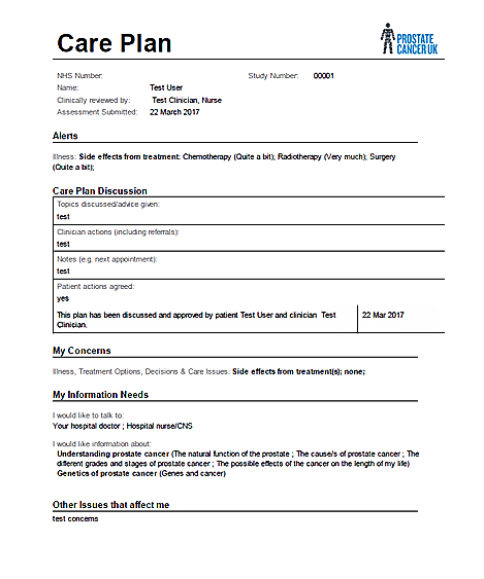
The care plan.

The care plan was developed through a series of versions following clinician feedback, which primarily emphasized the importance of brevity and easy identification of “red flag” items. The latter was seen by clinicians as representing clinical and psychological concerns that should prompt an urgent referral from the system to the nominated attending health care professional. The IT team created a clinician-facing website through which clinicians could access care plans associated with their patients. An example of the care plan is shown in Figure 2. The TrueNth CHAT-P project concluded in June 2016 following testing with patients and the finalization of the clinician-facing site that hosted the care plan. Although the findings were encouraging, an assessment of the impact of CHAT-P on consultation and ensuing actions would determine its potential in terms of care delivery. Funding for the next stage of the CHAT-P program, testing the impact of CHAT-P in the clinical setting, was obtained from the National Institute for Health and Care Research, Research for Patient Benefit program (grant PB-PG-0214-33092). This is briefly described in the subsequent section as an additional phase of work after the 3-stage development and testing process described earlier.

### Brief Description of CHAT-P Used in a Prospective Feasibility Study (2016-2018)

**Figure 3:**
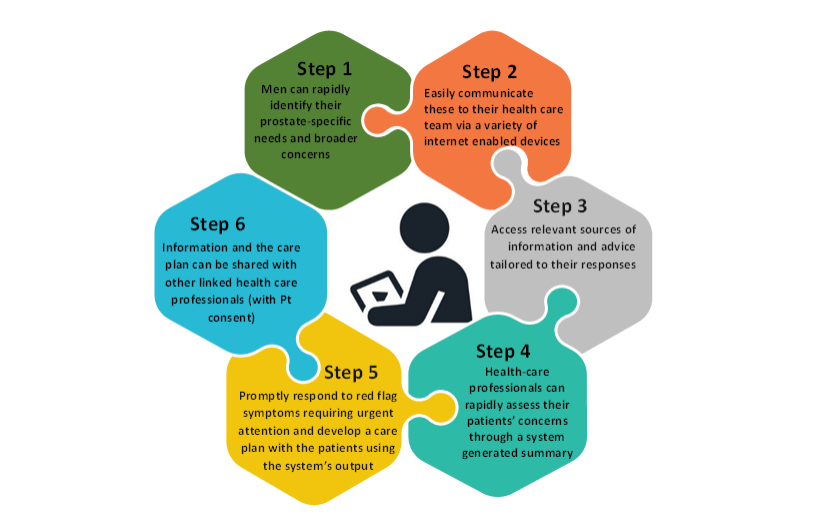
The potential of Composite Holistic Needs Assessment Adaptive Tool-Prostate in the prostate cancer care pathway.

We subsequently conducted a mixed methods feasibility study (Integrated Care in Prostate Cancer [36]) in a primary care setting with CHAT-P incorporated into the prostate cancer care pathway. In addition to testing the feasibility of a larger-scale study, this study provided a means of assessing the potential of the clinician-facing site and care planning function and the role of CHAT-P in enabling information sharing between patients and relevant clinical services. The information pathway is shown in Figure 3.

CHAT-P is built on a platform developed by a major information service provider, Infoflex-CIMS (now CIVICA), which is embedded in hospital trusts across the United Kingdom. Nevertheless, reaching an agreement regarding hosting and access to CHAT-P was challenging, requiring rigorous and complex processes involving the information services provider, NHS trust, and university undertaking the research. Governance approvals and penetration testing to ensure data security and agreements regarding data sharing were required before the start of the intervention.

Quantitative findings relating to the feasibility of the primary care–based study, patient use of CHAT-P, and technology acceptance and qualitative findings relating to patient and health care professional experience are reported in full elsewhere [36,37]. Several design issues were highlighted by the participants and by the practice nurses for attention in the final version. These are summarized in Multimedia Appendix 1.

The study demonstrated the practicality and utility of the assessment and improved information flow among the patient, primary care, and secondary care. The participants identified concerns in every domain of the assessment; emotional and psychological issues were the most commonly identified concerns, followed by physical problems and issues around access to services. Practice nurses followed up on these concerns and made appropriate onward referrals. When red flag symptoms were identified, immediate action was taken through phone calls to the secondary care team. The care planning document was easily accessible and straightforward to use. Primary care–based recruitment to the study proved challenging owing to the relatively small number of eligible participants identified in each participating practice and the competing demands on practice nurse time.

### Final Version

Following the feasibility study, the final version of CHAT-P is ready for implementation and evaluation at scale. The final version has incorporated the suggested changes, and major improvements have been made to the “Welcome” and “Introduction” screens. The welcome text has now been replaced by a short film featuring a patient, a GP, a CNS, and a consultant urologist (Multimedia Appendix 2). An overview of each element of CHAT-P is shown in cartoon format to prepare patients to access CHAT-P for the first time (Multimedia Appendix 3).

## Discussion

### Principal Findings

CHAT-P was developed in response to a need identified by men with prostate cancer and was coproduced with them, initially as part of the TrueNth program [30]. It aims to boost the agency and empowerment of men by enabling the identification and communication of their concerns as they change over time and by providing links to relevant information and advice. CHAT-P also seeks to help health care professionals provide optimum care by focusing consultations according to men’s individual, preidentified needs and to facilitate the provision of remote care, limiting the need for outpatient attendance.

There has been a rapid expansion in the field of electronic patient-reported outcome measures for people with cancer in recent years, and evaluation has shown considerable benefits [1]. HNA has also gone on the web; however, to date, these tools are generic, not adaptive, and, importantly, do not generate alerts in response to the selection of a red flag item [38]. As far as we have been able to ascertain, CHAT-P is the first web-based, adaptive tool that allows patients to select items from a series of menus and submenus without the need to respond to every item presented. CHAT-P is also unique in the wide-ranging nature of the 11 domains included and its ability to generate links in response to item selection.

### Technical Challenges

Initial challenges lay in ensuring that the content was both adequately holistic, including sufficiently detailed items within each of the 11 domains, and adequately specific to the needs of men with prostate cancer at different stages clinically. Question wording and response format had to be easily understood and consistent without being overly repetitive. The lack of clarity of language has hitherto been identified as a barrier to the uptake of web-based symptom monitoring [39,40]. Thus, we felt that coproduction with men with prostate cancer was critical to ensuring that CHAT-P met this key requirement.

Among the design challenges was the need to create a system that was both secure and easily accessible to men through registration and log-in process. A second challenge lay in developing a novel hierarchical branching system to ensure that top-level broad concerns led to relevant lower-level menus of items. Coding relevant links to sources of information and advice to appear in response to the selection of specific items was also a critical part of the design process. An additional issue for the team was to develop a care plan function, which was easy to use and fitted in with the current care pathway, importantly including the “red flag” items to alert both patients and health care professionals to serious concerns.

Through the iterative processes in the 3-stage development, we addressed each of these challenges and made amendments to the CHAT-P system, which were acceptable to the men with prostate cancer and their clinicians who were working with us.

### Strengths

Good communication and information tailored to patients’ concerns are the cornerstones of patient-centered care and underpin the concept of HNA. Both are central to the ability of people with cancer to manage the uncertainty of their situation, without which anxiety and depression frequently result [41]. CHAT-P has been coproduced by a team with a wide range of expertise, including men with prostate cancer, from the project planning stage. Their guidance has helped ensure that CHAT-P is accessible, comprehensive, and relevant to men’s needs. The iterative process of user testing and refinement has further contributed to its robust design. In the clinical setting, testing has demonstrated the wide-ranging concerns across domains that may be captured by an individualized adaptive assessment. In addition, testing has demonstrated the potential of the CHAT-P for information sharing between patients and clinical teams across settings.

In our study, men with prostate cancer were the key codrivers of the initial concept. The technology development and exploratory and feasibility studies have all involved a range of professional and lay stakeholders as team participants. Through the identification of common goals, close collaboration, and communication, differences in perspectives and requirements were acknowledged and discussed and compromises were reached that could combine end-user priorities, usability, and technical feasibility.

### Limitations

CHAT-P is built on a flexible platform that can be adapted to local requirements. Although this represents a strength, it also necessitates some active involvement of NHS IT departments and a member of the clinical team (eg, a CNS), who will also be required to monitor and update the links provided by the system.

An age-related digital divide in the use of IT is recognized as a barrier to the uptake of digital health technology. Prostate cancer is a disease that affects men increasingly as they age, and the risk of digital exclusion due to age was of particular concern in the early stages of the project. Our patient representative steering group members and project team members were all men with experience and confidence in IT, who were hence more inclined to see the usefulness and potential of a digital HNA. However, they were closely involved with other men with a range of IT skills whose views were also represented. In our interview study, all but one participant owned a computer tablet, and their computer literacy varied among low, medium, and high. Since the study, recently published figures indicate that 93% of the households in the United Kingdom now have access to the internet [42]. Although our sample represents a relatively narrow sociodemographic, it is an indication of a rising trend and suggests increasing familiarity with the digital world in the general population. In addition, the COVID-19 pandemic has seen a huge increase in the implementation and use of web-based health care and remote communication [43,44].

While our user testing involved men recruited through support associations, our patient participants in the interview study and in the subsequent feasibility study were sampled through clinic and general practice lists and represented a wider range of socioeconomic backgrounds; however, it is important to acknowledge that there was little ethnic diversity in our patient sample [36].

### CHAT-P in the Era of the COVID-19 Pandemic

Although the NHS has been championing the use of digital technology since 2012, remote consultation as a substitute for outpatient and general practice appointments has rapidly become established since the beginning of the COVID-19 pandemic. Overall, rates of remote consultations across general practice and hospital settings increased significantly due to the COVID-19 pandemic [45]. Remote consultation represents a considerable cost saving to the NHS, and where quality and safety criteria as advised by the General Medical Council are met, it is likely to become embedded as a part of the standard of care [46]. An initial driver for the development of CHAT-P was the notion that a cancer-specific HNA, combined with a PSA tracker, would enable remote monitoring for men with low-risk prostate cancer. Linked to telephone or video consultations, CHAT-P, which is able to identify and alert patients and clinicians to symptoms requiring urgent attention, may contribute significantly to the confidence of using remote care for men regardless of their disease profile and clinical pathway.

### Implementation and Evaluation

HNAs for all patients with cancer have been recommended in the recent NHS long-term plan, in addition to a personalized care plan and information to help overall health and well-being [47]. Evidence indicates that HNA is well accepted by patients. However, previous research has shown that generic HNAs (often without any clear link to a care planning consultation) have little impact on outcomes in terms of onward referrals or advice regarding self-management. Unless the HNA is used during consultations and incorporated into care plans and appropriate referrals made, its impact on patients’ quality of life can be negative because of expectations among patients being raised but remaining unmet, as a previous work by members of our team has demonstrated [17].

CHAT-P represents an advance in generic and especially paper-based HNA in terms of its potential to empower men to identify and communicate their changing needs over time and along the pathway of care and to provide reliable information and resources for men to actively manage their own health. However, its value must ultimately be judged in terms of its outcomes. A full evaluation of CHAT-P is needed of both process and outcomes to determine the extent and level of adoption in secondary care, as well as clinical, quality of life and health economic outcomes from the beginning of the clinical pathway. Recruited patients should be diverse in terms of both clinical and sociodemographic characteristics. Crucially, a theoretically driven implementation strategy is required. Evidence indicates that in the absence of such a strategy, digital innovations in health care are unlikely to succeed. The normalization process theory [48-51] has been used as the basis for the successful implementation of digital health innovations and may provide a useful framework for the development of implementation and adoption strategies in relation to CHAT-P and its evaluation. Awareness raising and training for staff on the use of the system and on how to introduce it to patients and encourage them to use it are critical elements of the implementation process. It is also important that staff do not perceive the intervention as a burden. A project to electronically capture and remediate the late effects of pelvic radiation reported significant variation in uptake by patients across sites, which was largely attributed to differences in staff engagement and perceptions of burden [15].

In addition, information governance and related approvals must be obtained, which may present a barrier to adoption. Conclusions from an intervention that includes an electronic HNA and remote monitoring within the context of a supported self-management pathway recommend the appointment of a clinical champion to drive and oversee this process [31].

Once established, we aim to investigate the potential for integrating the CHAT-P output directly to the National Prostate Cancer Audit site and the Somerset Cancer Registry to aid health policy makers in determining priorities for follow-up and care.

### The Future of Cancer-Specific HNA

We are now developing a second cancer-specific HNA focused on the needs of people with bladder cancer (CHAT-B). Following the testing process, it is intended that both systems will be integrated within the clinical pathway from the point of diagnosis onward in several NHS trusts. Studies are needed to determine the extent of adoption and evaluate effectiveness in improving quality of life outcomes, enhancing patient enablement, and reducing the demand for NHS resources.

### Conclusions

CHAT-P is the first web-based interactive platform for cancer-specific HNA. This platform provides an innovative means to allow men to communicate their concerns to their health care teams before an in-person or remote consultation. There is a need for a full evaluation of the implementation process and outcomes following the introduction of CHAT-P into the clinical pathway. The CHAT-P model also has the potential to be adapted to other cancers.

## Appendix

Multimedia Appendix 1 Final edits to Composite Holistic Needs Assessment Adaptive Tool-Prostate.

Multimedia Appendix 2 Composite Holistic Needs Assessment Adaptive Tool-Prostate film featuring clinicians, one of our patients, and representatives of public involvement.

Multimedia Appendix 3 Composite Holistic Needs Assessment Adaptive Tool-Prostate instruction video.

## Abbreviations

ADT: androgen deprivation therapy
CHAT-P: Composite Holistic Needs Assessment Adaptive Tool-Prostate
CNS: clinical nurse specialist
GP: general practitioner
HNA: Holistic Needs Assessment
PCUK: Prostate Cancer UK
PSA: prostate-specific antigen
