# Correction: A Web-Based Prostate Cancer–Specific Holistic Needs Assessment (CHAT-P): Multimethod Study From Concept to Clinical Practice

**DOI:** 10.2196/43856

**Published:** 2022-11-01

**Authors:** Veronica Nanton, Rebecca Appleton, Nisar Ahmed, Joelle Loew, Julia Roscoe, Radha Muthuswamy, Prashant Patel, Jeremy Dale, Sam H Ahmedzai

**Affiliations:** 1 Warwick Medical School University of Warwick Coventry United Kingdom; 2 Mental Health Policy Research Unit Division of Psychiatry University College London London United Kingdom; 3 Medical School University of Sheffield Sheffield United Kingdom; 4 Lucerne School of Business Lucerne Switzerland; 5 Prostate Cancer UK London United Kingdom; 6 Institute of Cancer and Genomic Sciences University of Birmingham Birmingham United Kingdom

In “A Web-Based Prostate Cancer–Specific Holistic Needs Assessment (CHAT-P): Multimethod Study From Concept to Clinical Practice” ([JMIR Cancer 2022;8(4): e32153]) the authors noted one error in the order of the authors.

The author list appeared as:

Rebecca Appleton, Veronica Nanton, Nisar Ahmed, Joelle Loew, Julia Roscoe, Radha Muthuswamy, Prashant Patel, Jeremy Dale, Sam H Ahmedzai

Whereas it should have been:

Veronica Nanton, Rebecca Appleton, Nisar Ahmed, Joelle Loew, Julia Roscoe, Radha Muthuswamy, Prashant Patel, Jeremy Dale, Sam H Ahmedzai

The correction will appear in the online version of the paper on the JMIR Publications website on 1^st^ November 2022, together with the publication of this correction notice. Because this was made after submission to PubMed, PubMed Central, and other full-text repositories, the corrected article has also been resubmitted to those repositories.

