# Supplementary material for: A Web-Based Prostate Cancer–Specific Holistic Needs Assessment (CHAT-P): Multimethod Study From Concept to Clinical Practice
Source: JMIR Cancer. 2022 Oct 19;8(4):e32153. doi: 10.2196/32153 (PMC9624375; doi:10.2196/32153)
Supplement: Multimedia Appendix 1 [file cancer_v8i4e32153_app1.docx]

## Appendix 1: Final Edits to CHAT-P

| **Themes** | **Suggested Actions** |
| --- | --- |
| **Look & Feel** | Patient Portal   - Enlarge and highlight ‘submit button’ on assessment pages. - Reduce size of logos on the 1^st^ screen and end of assessment screen pages. - **Rationale:** For ease of identification and consistency across the patient portal. Plus making the instructions text on the opening screen much easier to read. |
| **Functionality** | Patient Portal   - Consider password login quickly showing password characters as entered. - Ensure ease of registration and login, plus uploading time is tested across different types of online devices. - **Rationale:** To check loading time of website on each device is timely and users can check they are making the correct password entry. |
| **Content** | Patient Portal   - Change the text in red alert boxes to say 5 days rather than 7 days. - **Rationale:** to shorten patient response time for seeking assistance. - Enlarge the ‘Welcome’ banner on first screen, move central to screen & increase its visibility time. - **Rationale:** The welcome banner was missed by users due to its size, location on screen and short visibility time. - For consistency, rename ‘change responses’ button at the end of each summary page to ‘review response’s’. - Review introduction page and consider ways of reducing the amount of explanatory text. - **Rationale:** For user friendliness and ease of explanation. |
| **Information Links** | Patient Portal   - Enlarge text in further information links. - **Rationale:** as these are small and difficult to read. - Continue to review links on a regular basis to keep them relevant and up to date. |
| **Any other comments** | Clinicians Portal   - Simplify the steps to copy the completed care plan from CHAT-P into a patients’ notes. - **Rationale:** Very important that the care plan can exported into the individual patient notes as a whole document, in both hospital and GP settings. |
