# Supplementary figures and images for: A Web-Based Prostate Cancer–Specific Holistic Needs Assessment (CHAT-P): Multimethod Study From Concept to Clinical Practice
Source: JMIR Cancer. 2022 Oct 19;8(4):e32153. doi: 10.2196/32153 (PMC9624375; doi:10.2196/32153)

## Slide 1
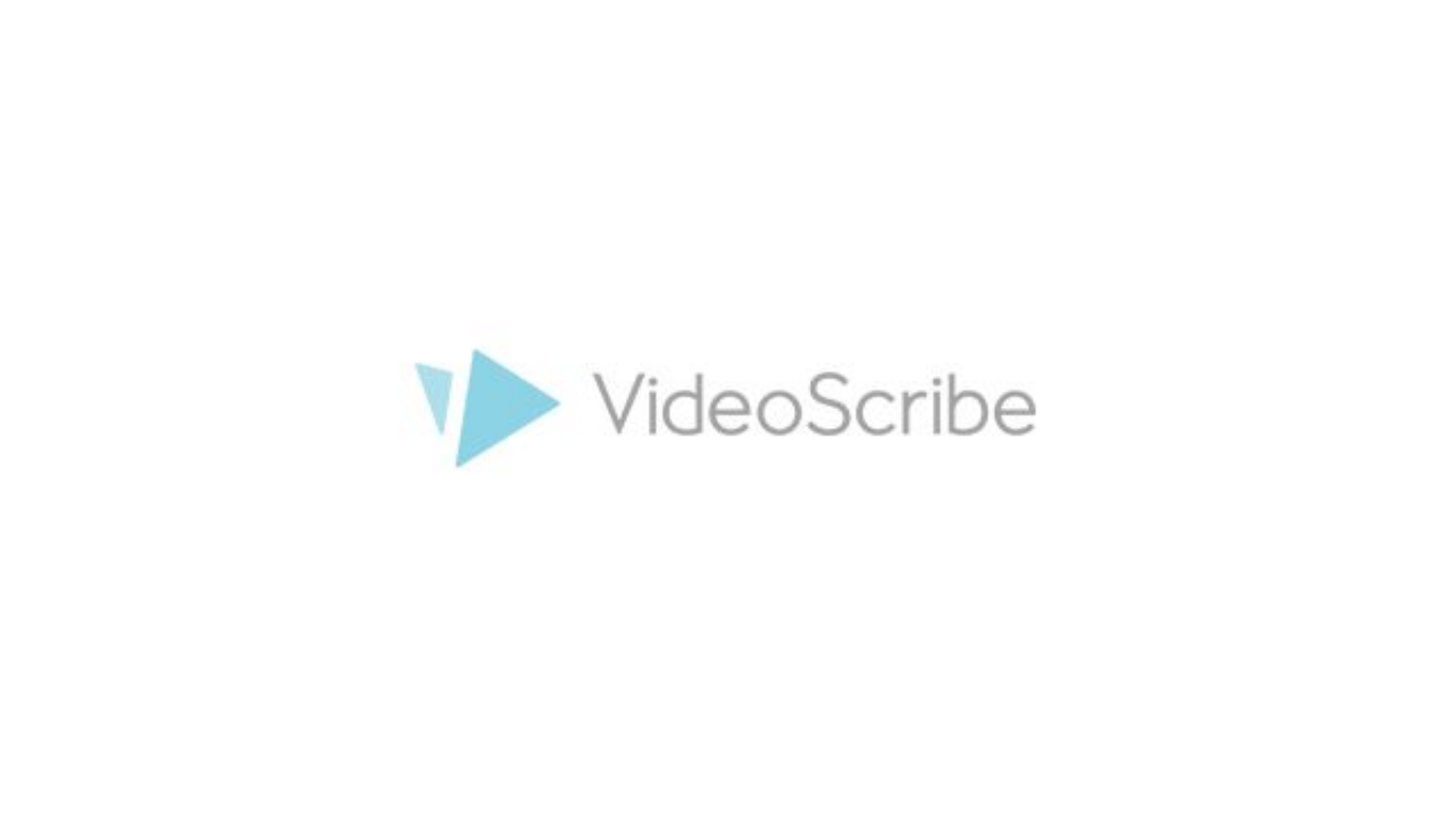

Supplement: Multimedia Appendix 3 [file cancer_v8i4e32153_app3.pptx]
